# Ebi3 Prevents *Trypanosoma cruzi-*Induced Myocarditis by Dampening IFN-γ-Driven Inflammation

**DOI:** 10.3389/fimmu.2017.01213

**Published:** 2017-09-26

**Authors:** Tiago Silva Medina, Gabriela Gonçalves Oliveira, Maria Cláudia Silva, Bruna Araújo David, Grace Kelly Silva, Denise Morais Fonseca, Renata Sesti-Costa, Amanda Farage Frade, Monique Andrade Baron, Barbara Ianni, Alexandre Costa Pereira, Christophe Chevillard, Edécio Cunha-Neto, José Antonio Marin-Neto, João Santana Silva

**Affiliations:** ^1^Ribeirão Preto Medical School, University of São Paulo, Ribeirão Preto, Brazil; ^2^Medical School/Heart Institute, University of São Paulo, São Paulo, Brazil; ^3^Department of Bioengineering, Brazil University, São Paulo, Brazil; ^4^Aix-Marseille Université, INSERM, GIMP UMR_S906, Marseille, France

**Keywords:** Ebi3, interleukin-27, myeloid cells, IFN-γ, Th1 lymphocytes, myocarditis, *Trypanosoma cruzi*, Chagas disease

## Abstract

The identification of anti-inflammatory mediators can reveal important targetable molecules capable of counterbalancing *Trypanosoma cruzi*-induced myocarditis. Composed of Ebi3 and IL-27p28 subunits, IL-27 is produced by myeloid cells and is able to suppress inflammation by inducing IL-10-producing Tr1 cells, thus emerging as a potential candidate to ameliorate cardiac inflammation induced by *T. cruzi*. Although IL-27 has been extensively characterized as a suppressive cytokine that prevents liver immunopathogenesis after *T. cruzi* infection, the mechanisms underlying its effects on *T. cruzi*-induced myocarditis remain largely unknown. Here, wild-type (WT) and Ebi3-deficient animals were intraperitoneally infected with trypomastigotes of *T. cruzi* Y strain and used to evaluate the potential anti-inflammatory properties of Ebi3 during *T. cruzi* infection. The survival rates of mice were daily recorded, the frequency of inflammatory cells was analyzed by flow cytometry and inflammatory mediators were measured by ELISA, real-time PCR and PCR array. We reported that *T. cruzi*-induced myocarditis was prevented by Ebi3. Stressors mainly recognized by TLR2 and TLR4 receptors on myeloid cells were essential to trigger IL-27p28 production. In addition, Ebi3 regulated IFN-γ-mediated myocarditis by promoting an anti-inflammatory environment through IL-10, which was most likely produced by Tr1 cells rather than classical regulatory T cells (Tregs), in the heart tissue of *T. cruzi*-infected animals. Furthermore, *in vivo* IFN-γ blockade ameliorated the host survival without compromising the parasite control in the bloodstream. In humans, IL-27p28 was correlated with cardiac protection during Chagas disease. Patients with mild clinical forms of the disease produced high levels of IL-27p28, whereas lower levels were found in those with severe forms. In addition, polymorphic sites at Ebi3 gene were associated with severe cardiomyopathy in patients with Chagas disease. Collectively, we describe a novel regulatory mechanism where Ebi3 dampens cardiac inflammation by modulating the overproduction of IFN-γ, the *bona fide* culprit of Chagas disease cardiomyopathy.

## Introduction

Chagas disease affects more than 8 million individuals worldwide and it is an important cause of morbidity and mortality in Latin America. The most severe clinical form of this parasitic disease is the chronic Chagas cardiomyopathy (CCC). The pathogenesis of CCC is multifactorial, involving cardiac dysautonomia, microvascular disturbances, parasite-dependent myocardial damage, and immune-mediated myocardial injury (1).

*Trypanosoma cruzi-*induced heart inflammation is caused by parasite antigens recognized by innate immune sensors, such as toll-like receptors (TLRs) and inflammasomes, that lead to the activation and infiltration of inflammatory cells into the heart and ultimately promote local cytokine and chemokine production (2–4). To maintain the cardiac inflammation as an ultimate goal of destroying the parasite, Th1 cells release IFN-γ that promptly acts on the neighboring macrophages and cardiac cells, whose transcriptional program dynamics are altered to induce the production of nitric oxide (NO), a key molecule with trypanocidal properties (4, 5). Although Th1 lymphocytes are essential for controlling the cardiac parasitism, it may lead to chronic inflammation that ultimately results in tissue damage and dysfunction (6).

Classical regulatory T lymphocytes (Tregs) are known to protect the host by reducing cardiac damage and preventing its progression (7). Tregs dampen *T. cruzi*-induced myocarditis mainly through the release of anti-inflammatory cytokines or inhibitory interactions, such as PD-1/PDL-1 (8, 9) or CLTA-4/CD80/CD86 (10, 11). Given the current lack of targets for therapeutic interventions, unraveling the regulatory mechanisms that prevent *T. cruzi*-induced myocarditis constitutes a reasonable research aim for the adequate control of CCC.

Characterization of novel mediators with anti-inflammatory properties is urgently necessary to prevent CCC. IL-27, a heterodimeric cytokine composed of Ebi3 and IL-27p28 subunits (12, 13), signals through a receptor composed of IL-27Rα and gp130 subunits (13). IL-27 is mainly produced by macrophages (14) and dendritic cells (15) and is known to promote IL-10-producing Tr1 cells by phosphorylating STAT1 and STAT3 (16). Although Tr1 cells have been characterized by their oscillatory capacity to produce IFN-γ (17), such regulatory cellular subset predominantly releases IL-10 to suppress inflammatory conditions (18). Pieces of evidence reveal that IL-27 and IL-12 can act synergistically to upregulate Blimp1 in pre-committed Th17 cells, thus polarizing them to a Tr1-like subpopulation (19). In addition, IL-27 can directly inhibit the transcription factors GATA3 (20) and RORγT (21), thus suppressing Th2 and Th17 lymphocytes. IL-27 also antagonizes T cell production of IL-2, a soluble factor required to Th1 maintenance (22).

The impact of IL-27 on the liver inflammation in both *T. congolense* (23) and *T. cruzi* (24, 25) infections has been assessed. Using IL27Rα^−/−^ (23, 24) or Ebi3 ^−/−^ (25) mice, IL-27 acts as a key suppressor of liver immunopathogenesis by inhibiting Th1 cells (23, 25) and their production of IFN-γ (23, 24). IL-27 signaling prevented damages in the hepatocyte architecture (23) and the emergence of necrotic areas in the liver (24, 25) of infected animals due to a local and systemic hyperproduction of inflammatory cytokines (23–25). Although IL-27 has been well characterized as a key inhibitor of liver inflammation caused by species of *Trypanosoma*, its role and implications to the outcome of *T. cruzi*-induced myocarditis is largely unknown. We hypothesized that Ebi3 is required for the induction of an anti-inflammatory environment and controlling of *T. cruzi*-induced myocarditis. Here, we showed that Ebi3 is essential to counterbalance the inflammatory environment induced in the heart of mice and humans due to the *T. cruzi* infection. Our results point out the importance of Ebi3 as a key suppressive molecule capable of inhibiting the deleterious inflammatory effects of IFN-γ, the *bona fide* culprit involved in the pathogenesis of Chagas disease cardiomyopathy.

## Materials and Methods

### Ethics Statement

Twenty-four patients were enrolled in this study after prior approval of the Institutional Ethics Committee (Hospital das Clínicas de Ribeirão Preto—USP, São Paulo, Protocol number 2285/2007; Brazil). Signed informed consent was obtained from all patients who were positive for at least two serology tests for Chagas disease, as determined by hemagglutination, immunofluorescence or ELISA techniques. Another cohort of 395 patients was used to study the importance of Ebi3 polymorphisms to the outcome of Chagas disease (Under the approval of the Institutional Ethics Committee—CAPpesq–HC-FMUSP—Protocol Number 0265/10, Brazil).

All the experiments using mice model were conducted with the approval of the Institutional Animal Care and Use Committee (IACUC)/Ethics Committee for Animal Care and Research (CETEA-FMRP/USP) under protocol 192/2011. The protocol was approved by the National Committee for Control of Animal Research (CONCEA).

### Patients

Based on a detailed clinical evaluation and the results of 12-lead rest electrocardiogram (EKG), chest X-ray, and a 2D-echocardiogram, such patients with Chagas disease were stratified into five clinical groups: asymptomatic, mild heart dysfunction, moderate heart dysfunction, and severe heart dysfunction. Blood samples were collected from the eligible patients and from seven healthy subjects to be used as controls. IL-27 production was evaluated in the plasma of patients using ELISA kit for the subunit IL-27p28. All the technical procedures were performed according to the manufacturers’ instructions. Results were expressed as nanograms per milliliter.

Another cohort of 395 patients was used to study the importance of Ebi3 polymorphisms to the outcome of Chagas disease. In this cohort, patients with Chagas disease were stratified into two groups based on the symptoms of the disease: patients with no symptoms were classified as indeterminate (asymptomatic) patients, whereas those with cardiac symptoms and complications were classified as CCC patients. CCC patients were further stratified based on their ejection fraction (EF): those with EF >0.4 were classified as patients having a moderate cardiopathy and those with EF ≤0.4 were classified as patients having severe cardiopathy. DNA of patients with Chagas disease was extracted, and probes specific for the SNPs rs4740 (Assay ID: C_2591150_20) and rs4905 (Assay ID: C_2591151_10) (Life Technologies) of Ebi3 were used.

### Animals, Parasites, and Experimental *T. cruzi* Infection

Mice aged 6–8 weeks were maintained in animal facilities at the Ribeirão Preto Medical School (FMRP-USP), housed with a 12 h light-darkness cycle and used to perform all experiments described in this study. We used female mice for all experiments comparing C57BL/6 (WT) mice with TLR2, TLR3, TLR4, TLR9, TRIF, MyD88, NRLP3, IL-17R, IL-23, IL-6, and Ebi3-deficient mice. All the experiments were conducted by using sex- and aged-matched mice. After approval by the Ethics Committee for Animal Care and Research of the FMRP-USP (CETEA-FMRP/USP, animal protocol 192/2011), animals were intraperitoneally infected with 1,000 trypomastigotes forms of *T. cruzi* (Y strain). To perform *in vitro* experiments, trypomastigotes forms were maintained in LLC-MK2 line (Rhesus Monkey Kidney Epithelial Cells).

### Mortality and Bloodstream Parasitemia

After infecting mice with the parasites, the survival rate was evaluated for 40 days post infection (d.p.i.). To quantify the bloodstream parasitemia, 5 µL of peripheral blood from infected animals were smeared on a 22 × 22 mm microscope slide, and the number of parasites determined. Parasitemia was analyzed from 5 to 15 d.p.i.

### PCR Array

Fragments with approximately 10 mg of heart tissues from WT and Ebi3-deficient mice were obtained from both non-infected and infected animals, 7 d.p.i. After collecting heart tissue of five animals per group, the samples were extracted, quantified, and examined by qPCR assay according to RT^2^ProfilerPCRArray™ kit of Mouse Innate Immune Responses and Adaptive (PAMM 052Z—Qiagen) on the ABI StepOnePlus™ equipment. RNA integrity was assessed by Agilent Bioanalyzer™ equipment following the manufacturer’s instructions. The non-normalized and normalized data were deposited in the Gene Expression Omnibus (GEO) database under the accession number GSE103330 (Link to the website over the accession number: https://www.ncbi.nlm.nih.gov/geo/query/acc.cgi?acc=GSE103330).

### Cardiac Parasitism and Damage

DNA from the heart, liver, spleen, and skeletal muscle of WT and Ebi3-deficient mice was purified using the SV Total DNA Isolation System kit (Promega, Madison, WI, USA) according to the manufacturer’s instructions. Real-time PCR was performed using the SYBR Green reagents (Invitrogen, Carlsbad, CA, USA) with 100 ng of total DNA. The sequences of primers used were TCZ-F 5′-GCTCTTGCCCACAMGGGTGC-3’ and TCZ-R 5′-CCAAGCAGCGGATAGTTCAGG-3′. The samples were amplified in a StepOnePlus Real-Time PCR System (Applied Biosystems, Foster City, CA, USA) with the following PCR conditions: first step (2 min at 50°C), second step (10 min at 95°C), and 40 cycles (30 s at 95°C, 15 s at 60°C, and 30 s at 72°C), followed by a dissociation stage. The results were based on a standard curve constructed with DNA from culture samples of *T. cruzi* trypomastigotes (*n* = 3). To determine heart and liver damage, the release of CK-MB (Creatine Kinase-MB), AST (Aspartate Aminotransferase), and ALT (Alanine Aminotransferase) into the bloodstream of normal and infected animals was measured using specific kit (Labtest^®^) following the manufacturer’s recommendations.

### *In Vitro* Culture of Splenocytes, Macrophages, and Dendritic Cells

To evaluate whether *T. cruzi* parasites were able to induce IL-27, 2 × 10^6^ splenocytes from normal WT mice were stimulated with 6 × 10^6^ trypomastigotes in 48-well plates. To identify cellular sources of IL-27, bone marrow-derived macrophages (BMDM) and bone marrow-derived dendritic cells (BMDC) were differentiated and cultured with *T. cruzi*. The BMDMs were obtained as described previously (26). Briefly, the total bone marrow cells were cultured in RPMI 1640 medium (Sigma-Aldrich), supplemented with 10% FBS (Invitrogen) and 30% of L-929 cell conditioned media or with 20% GM-CSF (PeproTech), and maintained at 37°C and 5% CO_2_ for 7 days. BMDMs and BMDCs were cultured with trypomastigotes of *T. cruzi* (MOI 3:1). After 7 h, splenocytes and *T. cruzi*-primed cells were collected for IL-27 mRNA quantification by real-time PCR and after 48 h the supernatants were collected for measuring IL-27. In order to evaluate which receptors and adapter molecules were involved in the production of IL-27, macrophages of WT and deficient mice of the molecules: NLPR3, TLR9, TLR3, TLR2, TLR4, TRIF, and MyD88 macrophages were differentiated according to the protocol above described. The ability of invasion of *T. cruzi* inside WT, TLR2^−/−^ and TLR4^−/−^ macrophages was evaluated after stimulation of cells with 10 ng/mL of IFN-γ followed by infection with trypomastigote forms of the parasite for 4 h. Then, macrophages were fixed and colored using panótico rapido^®^ according to manufacturer’s recommendations.

### Cytokine Quantification by ELISA

To assess the production of cytokines in the heart tissue of infected animals, myocardial fragments were collected into vials with a protease inhibitor cocktail (Complete, Roche), macerated, centrifuged, and the supernatant collected for cytokine quantification. The ELISA sets were IL-27p28, IL-10, TNF-α, IFN-γ, IL-4, and IL-13 (R&D, Minneapolis, MN, USA). All the technical procedures were performed according to the manufacturers’ instructions. The limits of sensitivity for the different assays were as follows: 15.6 pg/mL for IL-10 and IL-27p28; 31.2 pg/mL for TNF-α, IL-4 and IFN-γ; and 62.4 pg/mL for IL-13.

### Histological Analysis

Heart samples were fixed in 10% buffered formalin solution and, after 72 h, dehydrated in crescent concentration of ethanol solutions and embedded in paraffin. Blocks were sectioned at 5 thick and stained with hematoxylin-eosin (H&E). Using a light microscopy, the number of amastigote nests was counted in 25 images of each animal of the group, the inflammation was estimated with ImageTool 2.0 software (University of Texas Health Science Center, TX, USA).

### Real-time Quantitative PCR

Total RNA from cardiac tissue was isolated using TRIZOL (Invitrogen) and SV Total RNA Isolation System (Promega, Madison, WI, USA) according to the manufacturer’s instructions. cDNA was synthesized using 500 ng of RNA through a reverse transcription reaction (ImProm-IITM Reverse Transcriptase, Promega). Real-time PCR quantitative mRNA analyses were performed in a StepOnePlus Real-Time PCR System (Applied Biosystems) using SYBR Green reagents (Invitrogen) for quantification of the amplicons. The standard PCR conditions were as follows: 50°C (2 min), 95°C (10 min); 40 cycles of 94°C (30 s), 58°C (30 s), and 72°C (1 min); followed by a standard denaturation curve. Primers were designed by using the Primer Express software package v2.0 (Applied Biosystems), based on the nucleotide reference sequences available at GenBank database. Platinum SYBR Green qPCR SuperMix UDG with ROX reagent (Invitrogen), 1 mg/mL of each specific primer and a 1:20 dilution of cDNA were used in each reaction. The mean Ct values from duplicate measurements were used to calculate the expression of the target gene, with normalization to internal controls (GAPDH, β-actin, and HPRT).

### Intracellular Staining of Cytokines and Transcription Factors

Spleen and heart fragments were collected to evaluate IFN-γ-producing CD4^+^ T cells, IL-10-producing Tregs, and IL-10-producing Tr1 cells. To isolate mononuclear cells from cardiac tissues, the hearts were removed at 15 d.p.i. washed (to remove blood clots), minced with scissors into small fragments, extensively washed, and subjected to enzymatic digestion with 500 mg/mL of Liberase solution (Roche Applied Science, Indianapolis, IN, USA) for 1 h at 37°C. The tissues were washed with RPMI 10% FCS and total cells that passed through the cell strainer were subsequently counted. Two million leukocytes from heart tissue or from spleen were stimulated *in vitro* with PMA/Ionomycin/Golgi Stop for 6 h. The leukocytes were stained with specific antibodies for Cd11b (clone MI/70 BD), I-A/E (clone 269 BD), CD3 (clone 145-2C11), CD4 (clone RM4-5 BD), Foxp3 (clone MF 23 BD), IFN-γ (clone XMG 1.2 BD), IL-10 (clone JESS-16E3 BD), and IL-27p28 (clone MM27-7b1 Biolegend), and evaluated by flow cytometry.

### Neutralization of IFN-γ and Inhibition of iNOS

IFN-γ was neutralized *in vivo* by intraperitoneal injection of rat anti-mouse IFN-γ mAb (clone R4-6A2). The animals were treated with 2,000μg kg^−1^ of neutralizing IFN-γ antibodies at day 9, followed by 1,000μg kg^−1^ at days 12 and 15 d.p.i. To block NOS, animals were treated with 1,000 μg kg^−1^ of L-NMMA at 9, 12, 15, and 18 days d.p.i. Control mice were injected with equal quantities of vehicle. This delayed kinetics of blockade was chosen to allow an initial IFN-γ or NO response required to avoid uncontrolled parasite spreading and early mortality. After treatment, the mice were used for survival monitoring.

### Statistical Analysis

Data were expressed as means ± SEM. The Kaplan–Meier method was used to compare survival curves among groups. Student’s *t*-test was used to analyze the statistical significance of the observed differences in infected vs control assays, followed by Mann–Whitney post-test. In time course studies, one-way ANOVA was used, followed by Tukey–Kramer or Bonferroni *post hoc* analysis. All analyses were performed using PRISM 5.0 software.

## Results

### IL-17 Mediates Resistance to *T. cruzi* Infection and Attenuates the *T. cruzi*-Induced Cardiac Damage

**Figure 1:**
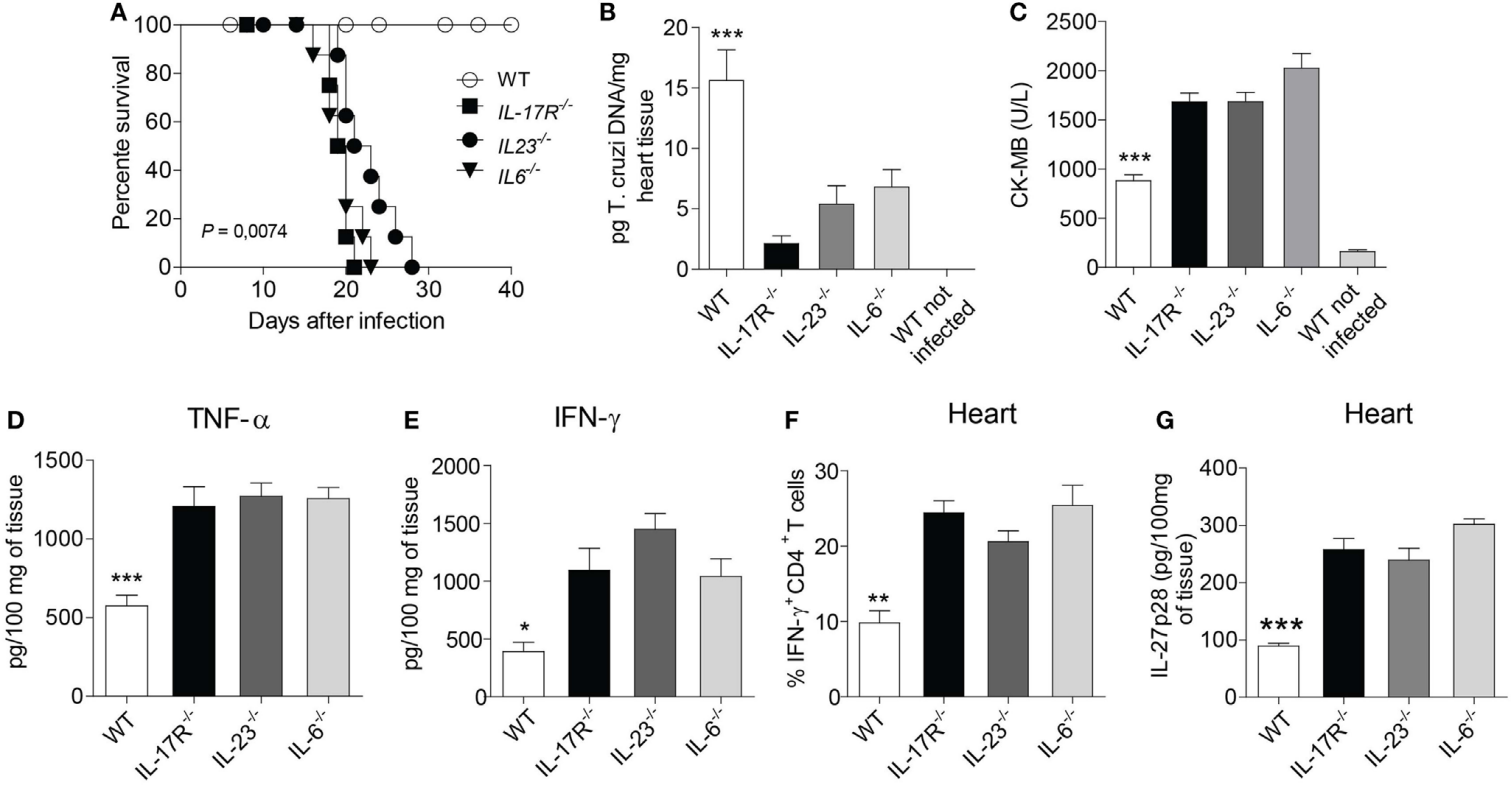
The absence of Th17-related molecules promotes intense recruitment of T_H_1 cells and tissue damage in the heart. **(A)** After infecting wild-type (WT), IL-17R-, IL-23-, and IL-6-deficient mice with 10^3^ trypomastigotes, the animal survival was daily assessed for 40 days. **(B)** Parasitism and cardiac damage were assessed by quantifying the parasite DNA with qPCR after 15 days of infection with *Trypanosoma cruzi*. **(C)** The serum levels of CK-MB were measured by commercial kits according to the manufacturer’s recommendations. **(D,E)** TNF-α and IFN-γ were measured by ELISA in the heart homogenate of infected WT and IL-17R-, IL-23-, IL-6-deficient mice after 15 days of infection. **(F)** The frequency of IFN-γ-producing CD4^+^ T cells in the heart tissue after 17 days of infection. Cells were obtained from heart, re-stimulated with PMA/Ionomycin for 6 h and subjected to intracellular staining for IFN-γ. Data were expressed as percentage of positive cells. **(G)** The production of IL-27p28 from 100 mg of heart tissue homogenate was assessed by ELISA. The data are representative of three independent experiments (*n* = 5). Data are expressed as mean ± SEM (*P* < 0.05).

Depending on the milieu, Th17 lymphocytes can be reprogrammed to Th1 lymphocytes (27). Upon *T. cruzi* infection, the IL-23–IL-17A axis was shown to be crucial for the maintenance of an optimal Th1 immune response (28). We have also previously reported that Th17 lymphocytes were protective cells during *T. cruzi* infection. Such cells antagonized the development of Th1 lymphocytes, thus protecting the hosts from Th1 cell-mediated myocarditis (29). Supported by these pieces of evidence, we used genetically deficient mice for Th17-related molecules to create an appropriate microenvironment that facilitate the polarization to Th1 lymphocytes after *T. cruzi* infection. Taking advantage of deficient mice allowed us to promote intense myocarditis characterized by a robust recruitment of Th1 lymphocytes in the myocardium of *T. cruzi*-infected mice. IL-17R-, IL-23-, and IL-6-deficient mice rapidly succumbed to the infection at 22–25 d.p.i. compared to mice of the control group (Figure 1A). Thus, although there is cardiac damage in the absence of Th17-related molecules, these animals could efficiently control the cardiac parasitism and minimize the consequent inflammatory changes as compared to the control group (Figure 1B). The strong inflammatory response in the absence of Th17-related molecules in the genetically deficient mice led to intense cardiac damage, as evaluated by increased serum CK-MB levels (Figure 1C). In addition, we found a robust production of TNF-α (Figure 1D), an intense recruitment of Th1 lymphocytes in the cardiac tissue in the absence of Th17-related molecules compared to WT counterparts at 17 d.p.i., as well as an increase in the IFN-γ production (Figures 1E,F). Also, the absence of Th17-related molecules led to a significant increase of IL-27p28 production (Figure 1G). These murine models prompted us to evaluate a possible regulatory function of IL-27 in this inflammatory condition. By contrast, the increase in IL-17 production in the absence of IL-27 signaling as expected was not observed (data not shown).

### Ebi3 Confers Resistance against *T. cruzi* Infection by Controlling Several Inflammatory Molecules in the Myocardium of Infected Mice

**Figure 2:**
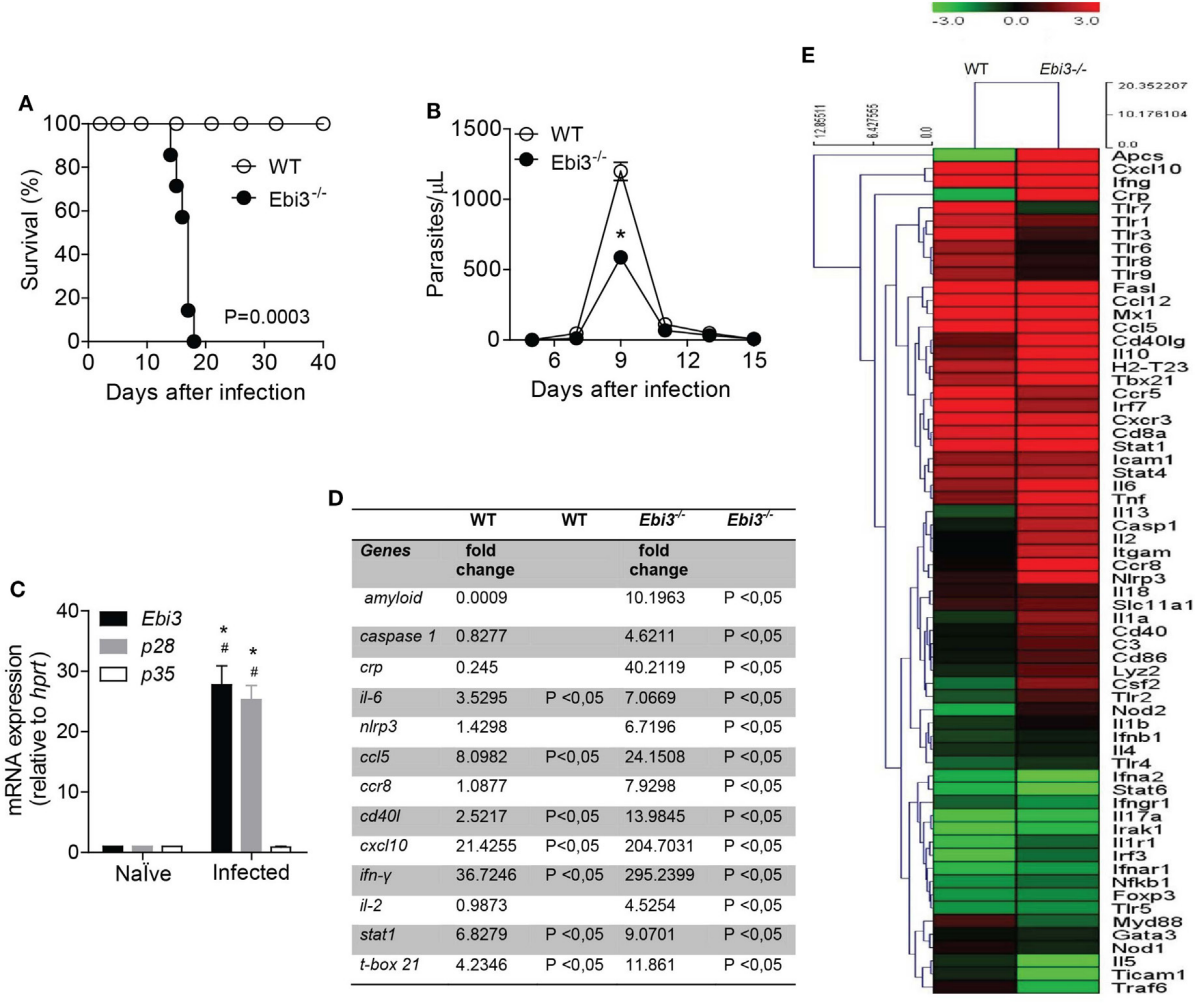
IL-27 is required for host protection by regulating several inflammatory molecules in the myocardium of *Trypanosoma cruzi*-infected mice. **(A)** The survival rate of wild-type (WT) and Ebi3-deficient mice infected intraperitoneally with 10^3^ trypomastigostes of *T. cruzi* was observed for 40 days. **(B)** The number of parasites in the bloodstream was counted from peripheral blood. Results are representative of three independent experiments (*n* = 8). Data are expressed as mean ± SEM (*P* < 0.05). **(C)** The mRNA expression from the heart tissue of Ebi3, p28, and p35 was evaluated at 9 d.p.i by real-time qPCR (*n* = 4). # *P* < 0.05 compared to naïve group. * *P* < 0.05 compared to expression of p35. **(D)** Most significant genes expressed 7 d.p.i with *T. cruzi* after analysis of gene expression by PCR array in heart tissue (data represented as fold change). **(E)** Hierarchical clustering showing gene expression in cardiac tissue of WT (*n* = 5) and Ebi3-deficient mice (*n* = 5) 7 days after infection with 10^3^ trypomastigotes of *T. cruzi* relative to non-infected mice (*n* = 5). The heat maps display the fold change expression of the genes. The color scale represents log2 transformation of % of reads. The transition of colors from green to red represents an average of lowest to highest fold change found (*P* < 0.05). The clustering method used was Euclidian distance with average linkage. Data are representative of two independent experiments.

IL-27 seems to be a key anti-inflammatory mediator in different inflammatory settings, including its relatively known role on the control of the liver immunopathology after *T. congolense* (23) or *T. cruzi* (24) infections and its suppressive effects on CD4 T cell subpopulations (25). Since CCC is induced and maintained by inflammatory CD4 T cell subpopulations, mainly Th1 cells, we evaluated whether IL-27 also mediates suppressive effects on the inflammation of the myocardium after *T. cruzi* infection. *In vivo* findings revealed that mice deficient for Ebi3, a subunit of IL-27, were extremely susceptible to infection with 10^3^ trypomastigote forms of *T. cruzi*, a quantity that was not lethal to WT mice (Figure 2A). These Ebi3-deficient mice died from *T. cruzi* infection within 15–16 d.p.i., whereas WT mice survived until 40 d.p.i. In addition, the bloodstream parasitemia levels in Ebi3-deficient mice were lower compared to WT mice, notably at the peak of the infection (9 d.p.i.) (Figure 2B). Since IL-35 is composed of the subunits Ebi3 and p35, we confirmed that IL-27 participates of the immune response during *T. cruzi* infection by assessing the expression of Ebi3, p28, and p35 in our model. In agreement with the importance of IL-27 to the *T. cruzi* infection, we observed that the gene expression of the subunits p28 and Ebi3 was higher than p35 at the peak of the parasitemia (Figure 2C). This finding does not rule out a potential participation of IL-35 upon *T. cruzi* infection.

IL-27 is thought to protect against tissue damage and regulate inflammatory conditions driven by effector T cells (23, 24). Based on the potential regulatory role of IL-27 upon inflammation, we screened the expression of inflammation-related genes in the heart tissue of WT and Ebi3-deficient mice after *T. cruzi* infection by performing a PCR array (Figures 2D,E). As expected, we found genes associated with markers of acute inflammatory response, such as serum amyloid P component (*Apcs*), C-reactive protein (*Crp*), and TNF-α (*Tnf*), that were highly expressed in the heart tissue of Ebi3-deficient mice compared to WT mice 7 d.p.i. *Casp1, Il6*, and *Nlrp3* genes were also upregulated in the cardiac tissue of Ebi3-deficient mice compared to WT mice. We also observed that Ebi3 was able to control the expression of chemokine-related genes along with the expression of the *Cd40lg* gene. The expression of *Ccl5, Ccr8*, and *Cxcl10* was increased in the heart of Ebi3-deficient mice compared to WT mice. We further explored whether Ebi3 downregulated effector T cell pathways. Although Ebi3 was not able to regulate Th2 lymphocytes-related genes, such as *Gata3, Stat6, Il4*, and *Il5*, we found that Th1 lymphocytes-related genes, such as *Il2, Stat1*, and *Tbx21*, were upregulated in the heart of Ebi3-deficient mice compared to WT mice. Of note, *Ifng* was one of the most strikingly expressed genes in Ebi3-deficient mice. The expression of *Foxp3* and *IL17a* was not changed 7 days after infection in both groups.

### Ebi3 Attenuates Myocarditis and Hepatic Damage in *T. cruzi*-Infected Mice

**Figure 3:**
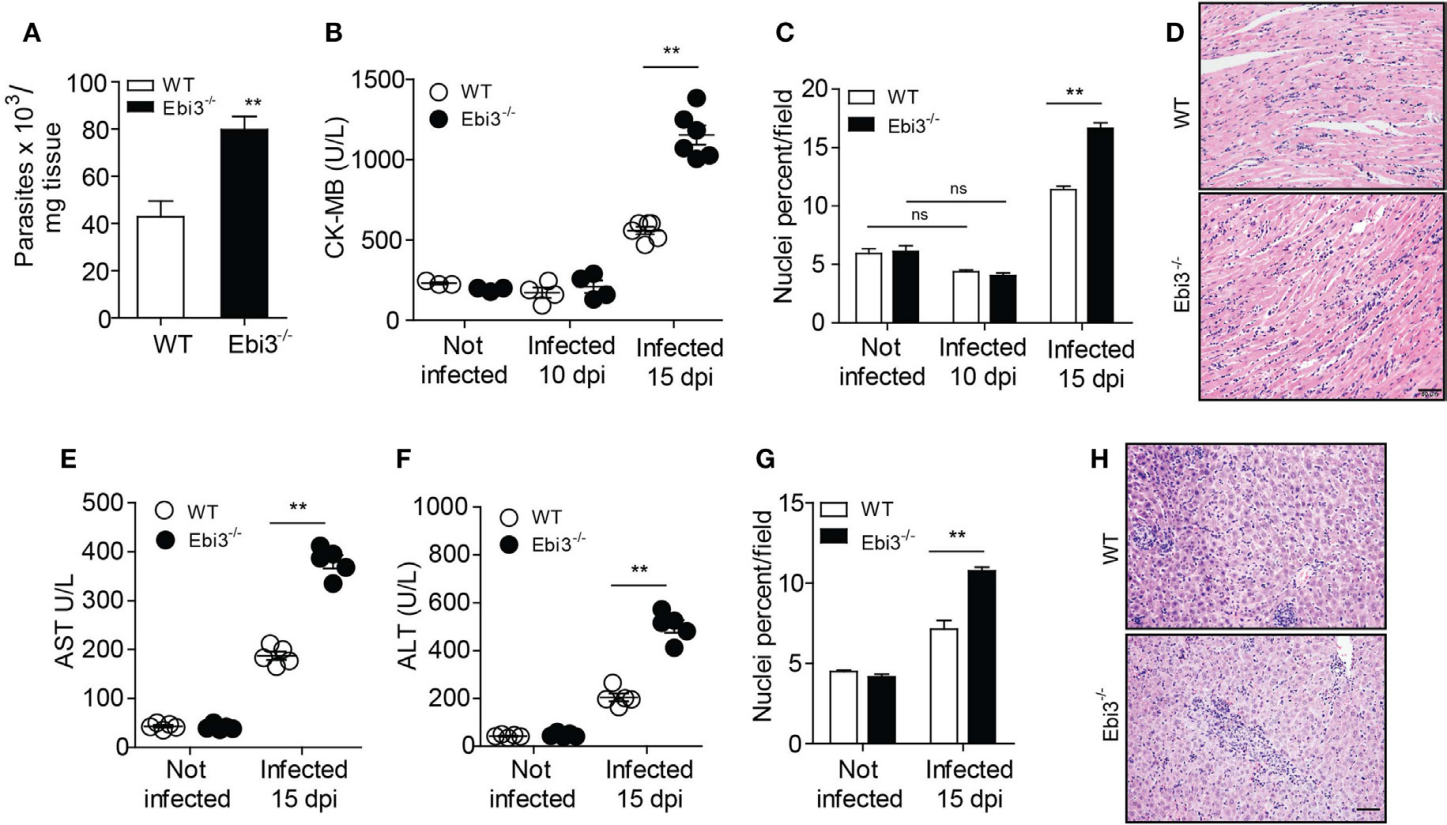
IL-27 is required to prevent cardiac parasitism and tissue damage after infection. **(A)** Parasitism in heart tissue of wild-type (WT) and Ebi3-deficient mice was assessed by qPCR and quantified by using a standard curve prepared with parasite DNA extracted from 1 × 10^7^ cultured *Trypanosoma cruzi*. qPCR was performed 15 d.p.i. with *T. cruzi*. **(B)** Heart damage was quantified by measuring CK-MB released into the blood. **(C,D)** Hematoxylin and eosin (H&E) staining of heart samples from infected wild-type (WT) and Ebi3-deficient mice. The images were taken and the inflammatory infiltrate was quantified using the *Image J* program at 200× magnification. Scale bar = 50 µm. **(E,F)** Serum AST and ALT levels were measured in WT and EBi3-deficient mice 15 days after infection. The enzymes were evaluated using commercial kits and the individual results expressed in U/L. **(G,H)** H&E staining of liver samples from infected WT and EBi3-deficient mice. The inflammatory infiltrate was quantified with the *Image J* program at 200× magnification. Scale bar = 50 µm. Data are expressed as mean ± SEM (*P* < 0.05) and are representative of two independent experiments (*n* = 5).

*Trypanosoma cruzi* parasites have preferential tropism for the skeletal muscle tissue. Locally, an enhanced recruitment of inflammatory cells is required to control and eliminate parasitemia and tissue parasitism, but it eventually culminates in nearly fatal *T. cruzi*-induced myocarditis (1). It was observed that Ebi3-deficient mice exhibited an increased heart parasitism (Figure 3A) with concomitant alongside increased blood levels of CK-MB at day 15 of infection, indicating intense myocardial damage (Figure 3B). We also observed a prominent cardiac inflammatory infiltrate in the myocardium of 15 day-infected Ebi3-deficient mice compared to their WT counterparts, although, as expected, the infiltrate is also higher in the 15-day-infected WT mice when compared to non-infected mice (Figures 3C,D). In addition, we found no myocardial damage (Figure 3B) or immune cell infiltration (Figure 3C) into the heart at day 10 post-infection, suggesting that the cardiac inflammation arises at the late time points and is highly destructive. The levels of AST and ALT were also assessed as indicators of liver damage (Figures 3E,F). The histology showed a lower inflammatory infiltrate in the liver of WT mice in comparison to Ebi3-deficient mice (Figures 3G,H), as previously shown elsewhere (23–25), suggesting that Ebi3 is also required to prevent liver immunopathology in *T. cruzi* infection. These results suggest that Ebi3 restricts the recruitment of inflammatory cells to the target tissues, besides controlling the *T. cruzi*-induced myocarditis. These data reveal that Ebi3 plays a crucial role for host survival and parasite control during the experimental *T. cruzi* infection.

### *T. cruzi* Induces Production of IL-27p28 by Myeloid Cells

**Figure 4:**
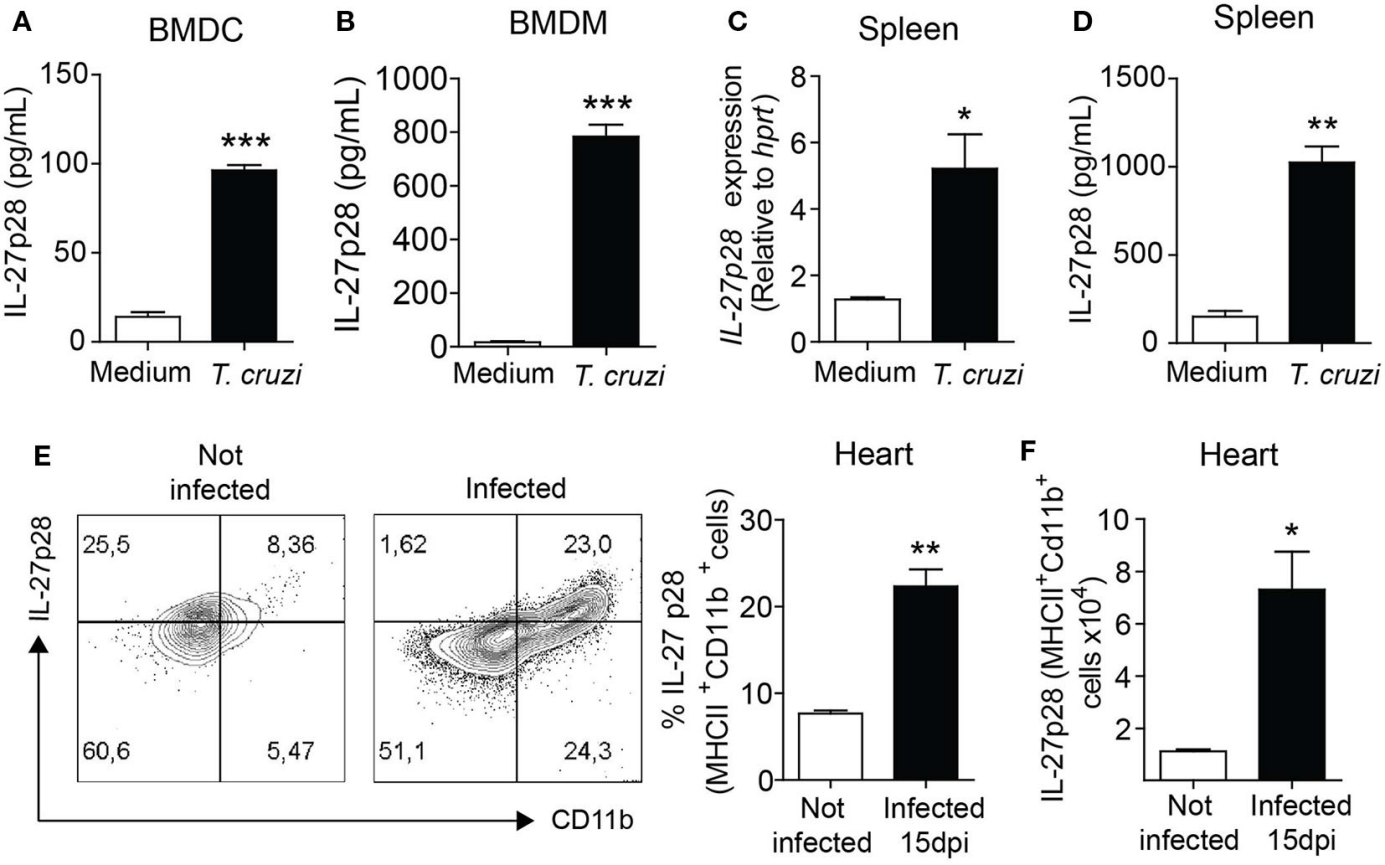
IL-27 is highly produced by myeloid cells following infection with *Trypanosoma cruzi*. **(A,B)** Dendritic cells and macrophages were differentiated from bone marrows of naïve wild-type (WT) mice and stimulated with medium or *T. cruzi* trypomastigotes (MOI 3:1). After 48 h, supernatant was collected and IL-27p28 levels were assessed by ELISA. **(C,D)** Splenocytes from naïve WT were cultured with medium or trypomastigotes (MOI = 3:1) for 6 or 48 h for mRNA or protein quantification of IL-27. **(E,F)** Intracellular expression of IL-27p28 by MHCII^+^CD11b^+^ myeloid cells 15 days after *T. cruzi* infection. Hearts of the WT mice infected or not were collected and digested with a cocktail of liberase TL (Roche). Cells were re-stimulated *in vitro* with PMA/ionomycin for 6 h and subjected to extracellular staining for MHCII and CD11b and intracellular staining for IL-27p28. Data are expressed as representative dot plots, percentage, and absolute number of IL-27^+^MHCII^+^CD11b^+^ cells. Data shown are mean ± SEM of cultured cells in triplicates and from five mice per group and are representative of two independent experiments.

IL-27 is thought to be preferentially produced by macrophages (14) and dendritic cells (15) to control inflammation. To identify the cell populations involved in IL-27p28 production, we analyzed their production after *in vitro* infection with *T. cruzi* parasites. We found that wild type (WT) BMDC infected with *T. cruzi* produced increased levels of IL-27p28 compared to the non-infected WT BMDC (Figure 4A). Macrophages are another essential cell source of IL-27. Similarly, *T. cruzi*-infected WT macrophages produced significantly higher levels of IL-27p28 than their non-infected WT counterparts (Figure 4B). We also found that IL-27p28 was strongly induced at both mRNA and protein levels by *in vitro T. cruzi*-infected spleen cells when compared to non-infected spleen cells (Figures 4C,D). Both macrophages and dendritic cells appear to be sources of IL-27p28 during the experimental *T. cruzi* infection. We also assessed IL-27p28 production in the myocardium of WT mice. We found a higher percentage and number of IL-27-producing MHII^+^CD11b^+^ myeloid cells in the myocardium of infected compared to non-infected mice (Figures 4E,F). These data suggest that myeloid cells are important sources of IL-27p28 under *in vivo* and *in vitro* stimulation with *T. cruzi*.

### *T. cruzi*-Induced IL-27p28 Production Is Mainly Dependent on TLR2 and TLR4 Receptors

**Figure 5:**
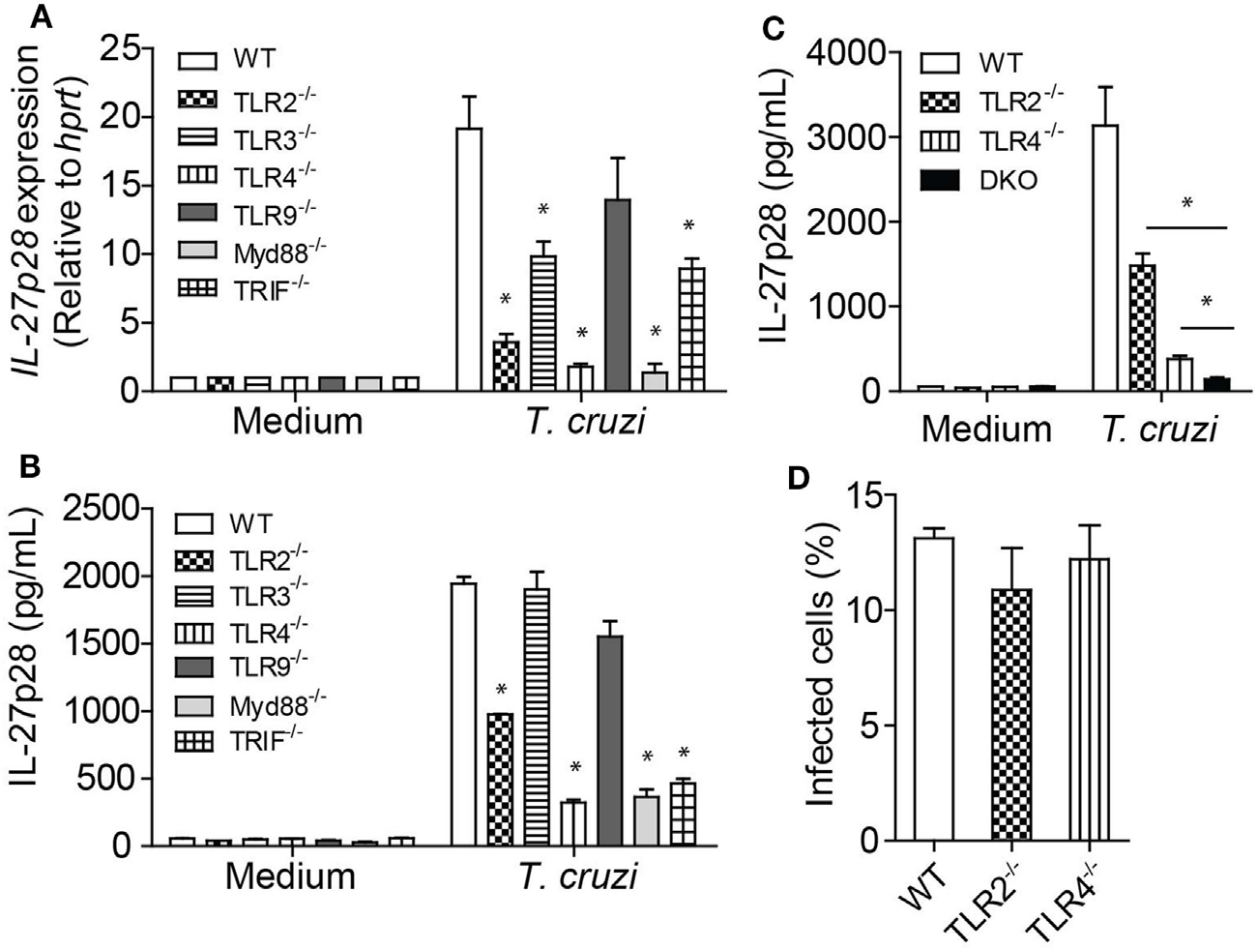
Innate immune receptors on macrophages are capable of recognizing *Trypanosoma cruzi* and induce IL-27p28 production. **(A,B)** Bone marrow-derived macrophages (BMDM) from wild type (WT) and deficient mice for the molecules *Tlr2, Tlr3, Tlr4, Tlr9, MyD88, and Trif* were infected with *T. cruzi* trypomastigotes (MOI 3:1). The mRNA expression of IL-27 was evaluated after 6 h by qPCR as well as the production of IL-27p28 after 48 h by ELISA. Amplified products were normalized in relation to *hprt* endogenous gene. **(C)** BMDM from WT and deficient mice for *Tlr2, Tlr4*, or *Tlr2/4* double knockout mice were infected with *T. cruzi* trypomastigotes (MOI 3:1) and the production of IL-27 was evaluated after 48 h by ELISA. **(D)** BMDM from WT and deficient mice for *Tlr2* or *Tlr4* were infected with *T. cruzi* trypomastigotes (MOI 3:1) and, after 4 h, the macrophages were fixed and colored with panotico rápido^®^ and the percentage of infected cells was measure. Results are representative of three independent experiments performed in quintuplicate. Data are expressed as mean ± SEM * (*P* < 0.05) compared to WT group.

Some innate immune receptors are known to recognize *T. cruzi*-expressed molecules or *T. cruzi*-derived danger signals, including TLRs 2 (30), 4 (31), and 9 (32), as well as NOD1 (33) and NLRP3 (34). It has earlier been reported that IL-27 production is also dependent on MyD88 and TRIF (35). Here, we assessed whether *T. cruzi* recognition by innate sensors could trigger IL-27p28 expression and production in macrophages. We found that *T. cruzi*-infected BMDM deficient for TLR2, TLR3, and TLR4 receptors, and MyD88 and TRIF adaptors had a lower expression of the gene *IL27p28* compared to BMDM of WT mice (Figure 5A). We also found that *T. cruzi*-infected BMDM deficient for TLR2, TLR4, MyD88, or TRIF had significant impairment of IL-27p28 production (Figure 5B). Since signaling through TLR2 and TLR4 had a strong impact on IL-27p28 production, we analyzed whether the lack of both receptors would abolish IL-27p28 production by macrophages. Indeed, the IL-27p28 levels produced by TLR2^−^TLR4^−^ (DKO) BMDM were much lower than by their WT counterparts and are equivalent to the basal levels produced by non-stimulated macrophages (Figure 5C). To prove that the IL-27p28 production was a direct effect of TLR2 and TLR4 signaling rather than a higher susceptibility to the *T. cruzi* infection caused by the absence of these receptors, we evaluated whether the macrophages were equally infected by the parasite regardless the expression of TLR2 or TLR4. As expected, the absence of TLR2 or TLR4 did not alter the susceptibility of the macrophage to the infection (Figure 5D), suggesting that the cellular signaling through these innate immune receptors, mainly TLR2 and TLR4, is required for IL-27p28 production during *T. cruzi* infection.

### Ebi3 Is Crucial to Prevent the Overproduction of IFN-γ in the Heart of *T. cruzi*-Infected Animals

**Figure 6:**
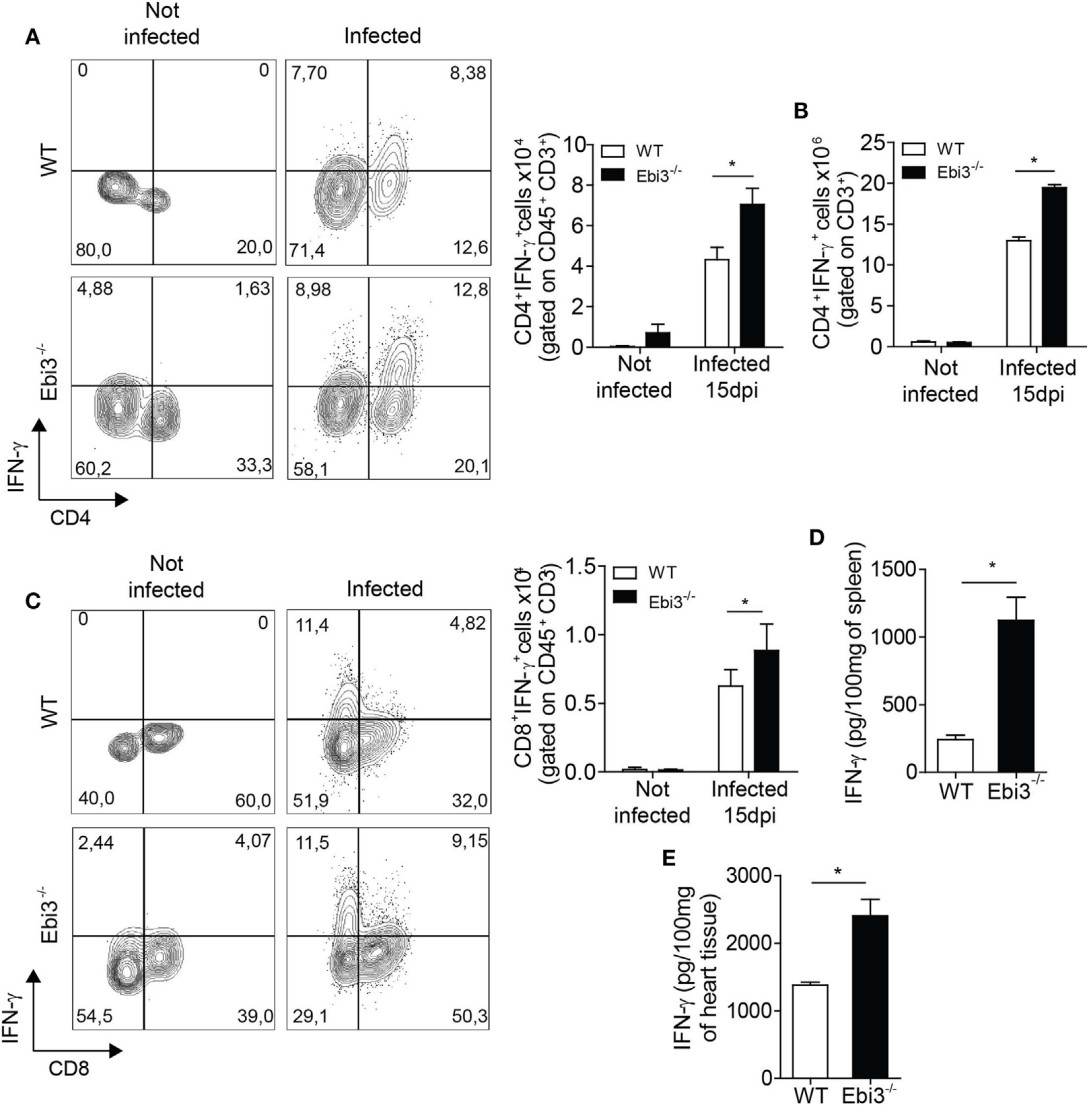
Inflammation in the spleen and heart of Ebi3-deficient mice is characterized by increased number of IFN-γ-producing T cells and higher secretion of IFN-γ. **(A–C)** Number of CD3^+^CD4^+^IFN-γ^+^ or CD3^+^CD8^+^IFN-γ^+^ infiltrating cells in the heart and spleen of wild-type (WT) and Ebi3-deficient mice. Cells from heart and spleen of WT and Ebi3^−/−^ naïve mice or infected with 10^3^ *Trypanosoma cruzi* tripomastigotes after 15 d.p.i. were stimulated *in vitro* with PMA/Ionomycin for 6 h and stained with anti-CD3 and anti-CD4 or anti-CD8 antibodies followed by intracellular staining with anti-IFN-γ antibody. **(D,E)** Production of IFN-γ in the homogenate of spleen and heart tissue 15 d.p.i. was quantified by ELISA. Data shown are expressed as mean ± SEM *(*P* < 0.05) and are representative of three independent experiments (*n* = 5).

We next explored whether Ebi3 could antagonize Th1 cell-induced inflammatory reactions after *T. cruzi* infection. We found increased number of IFN-γ producing CD4^+^ T cells in the cardiac tissue and spleen of Ebi3-deficient mice compared to WT counterparts 15 days after *T. cruzi*-infection (Figures 6A,B). In addition, number and frequency of IFN-γ-producing CD8 T cells were also higher in infected Ebi3-deficient mice compared to the control group. Although CD8^+^ T cells are also important to control the heart parasitism, the main source of IFN-γ into the heart of infected mice was CD4 T cells instead of CD8 T cells (4.32 × 10^4^ × 0.62 × 10^4^ in WT and 7 × 10^4^ × 0.88 × 10^4^ in Ebi3^−/−^ mice) (Figures 6A,C). In accordance with increased number of IFN-γ producing cells, we also found higher levels of IFN-γ in spleen (Figure 6D) and cardiac tissue (Figure 6E) of 15-day-infected Ebi3-deficient animals when compared to WT group. These data suggest that Ebi3 is a key regulator of IFN-γ production during the *T. cruzi*-induced myocarditis.

### Ebi3 Is a Key Modulator of the Th1 Cell-Coordinated Myocarditis by Promoting Regulatory IL-10-Producing Tr1 Cells

**Figure 7:**
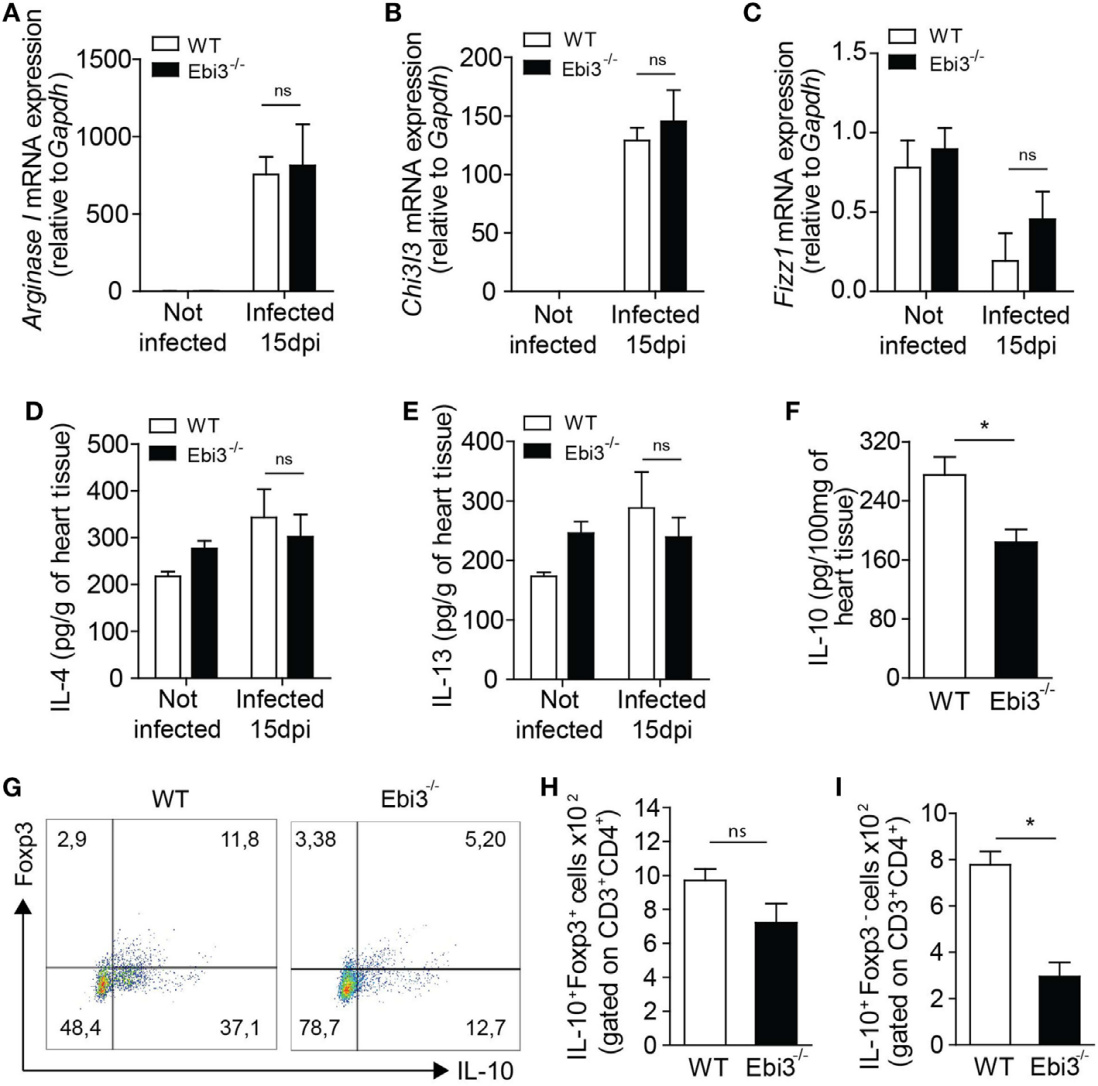
Cardiac inflammation of Ebi3-deficient mice is depicted by lower infiltration of IL-10-producing Tr1 cells. **(A–E)** Expression of key markers associated with alternatively activated macrophages was analyzed in the heart tissue of wild-type (WT) and Ebi3-deficient mice after 15 d.p.i. Gene expression of *ArgI* **(A)**, *Chi3l3* **(B)**, and *Fizz1* **(C)** was assessed by RT-PCR. IL-4 **(D)** and IL-13 **(E)** production was measured in the homogenate of heart tissue by ELISA. **(F)** Production of IL-10 was evaluated by ELISA in the homogenate of heart tissue 15 d.p.i. **(G,H,I)** Representative plot and number of CD3^+^CD4^+^IL-10^+^Foxp3^+^ and CD3^+^CD4^+^IL-10^+^Foxp3^−^ cells isolated from the heart tissue and evaluated as in **(A,B)**. Data shown are expressed as mean ± SEM *(*P* < 0.05) and are representative of three independent experiments (*n* = 5).

We next assessed whether Ebi3 was indirectly suppressing Th1 cells by inducing a regulatory cell population. We firstly assessed whether Ebi3 could be regulating alternatively activated macrophages. Although the infection upregulated the gene expression of markers expressed on alternatively activated macrophages (*Arg1* and *Chi3l3*), Ebi3 did not have any impact on their expression in the heart of 15-day-infected animals (Figures 7A,B). Also, *Fizz1* was downregulated in the heart of both groups after the infection with *T. cruzi* (Figure 7C). Moreover, IL-4 and IL-13, key cytokines associated with M2 macrophages, were not modulated by the infection or the absence of Ebi3 in the hearts of animals (Figures 7D,E). Taken together, these data suggest that Ebi3 cannot regulate key molecules expressed on alternatively activated macrophages in the heart tissue of *T. cruzi*-infected mice. We also evaluated whether Ebi3 could promote classical Tregs and/or Tr1 cells during the infection. We found that IL-10 production was significantly reduced in the myocardium of Ebi3-deficient mice compared to WT mice at day 15 after infection (Figure 7F). Unexpectedly, the number of classical CD4^+^Foxp3^+^ Tregs producing IL-10 was similar in the cardiac tissue of Ebi3-deficient and WT mice at day 15 after infection (Figures 7G,H), suggesting that Ebi3 was unable to promote the development of classical Tregs. By contrast, the frequency of IL-10-producing CD4^+^Foxp3^−^ T cells was significantly reduced in the myocardium of Ebi3-deficient mice compared to WT mice (Figures 7G,I), suggesting that the main cellular source of IL-10 production induced by IL-27 were CD4^+^Foxp3^−^ T cells (Tr1 cells). These observations show that Ebi3 could mediate Tr1 cell induction in the heart tissue of *T. cruzi*-infected animals.

### Blockade of IFN-γ or NOS Prevents Myocarditis and Ameliorates the Survival of *T. cruzi*-Infected Ebi3^−/−^ Mice

**Figure 8:**
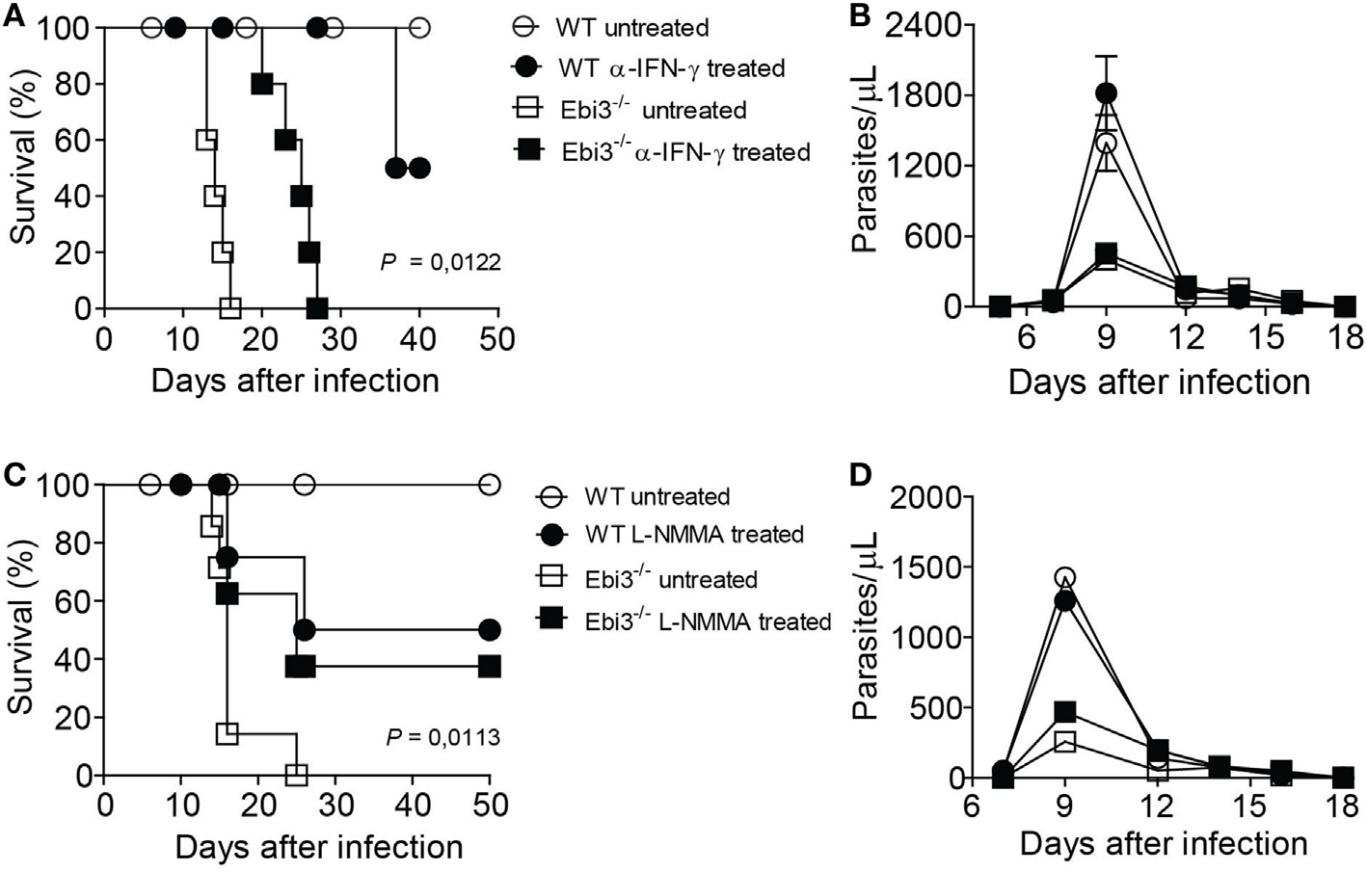
Blockade of IFN-γ or NOS prevents host susceptibility of *Trypanosoma cruzi*-infected Ebi3-deficient mice. **(A–D)** Ebi3-deficient or wild-type (WT) mice previously infected with *T. cruzi* were treated with α-IFN-γ at 9, 12, and 15 d.p.i. or L-NMMA at 9, 12, 15, and 18 d.p.i. intraperitoneally. **(A,C)** Mortality was daily evaluated and **(B,D)** bloodstream parasitemia was analyzed on alternate days from 5th until 18th day after infection. These data are representative of three independent experiments (*n* = 5) and expressed as mean ± SEM (*P* < 0.05).

Based on our earlier findings that Ebi3 could attenuate inflammatory intensity by inducing Tr1 cells after *T. cruzi* infection, we evaluated whether the treatment of *T. cruzi*-infected Ebi3-deficient mice with anti-IFN-γ MAb could control myocarditis and improve host survival. Notably, the blockade of IFN-γ after 9 d.p.i. was essential to improve host survival of Ebi3-deficient mice when compared to untreated Ebi3-deficient mice (Figure 8A). Because IFN-γ is required for parasite control by promoting macrophage release of NO, we assessed whether the blockade of IFN-γ contributed to *T. cruzi* growth. The treatment with α-IFN-γ MAb did not influence the level of parasitemia (Figure 8B). As IFN-γ also promotes NO production of macrophages, we assessed whether the use of L-NMMA to blockade all isoforms of NOS could similarly prevent myocarditis during the *T. cruzi* infection. As expected, treatment with L-NMMA was also able to prevent host susceptibility to *T. cruzi* infection of Ebi3-deficient mice (Figure 8C). Furthermore, treatment with L-NMMA after 9 d.p.i. did not lead to impaired *T. cruzi* clearance from the bloodstream (Figure 8D).

### Augmented Serum Levels of IL-27p28 Are Associated with Milder Clinical Forms of Chagas Disease

**Figure 9:**
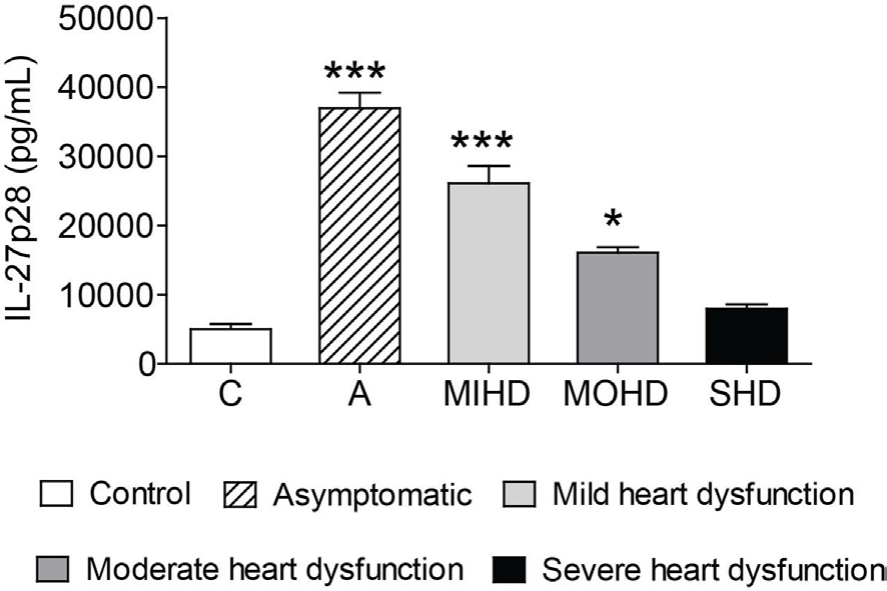
Increased serum levels of IL-27 are related to milder clinical forms of chronic chagasic cardiomyopathy. IL-27p28 was measured in the sera of control individuals, patients with indeterminate form, or patients with mild, moderate, or severe heart dysfunction stratified by clinical evaluation and criteria for Chagas disease further specified in the Section “Materials and Methods.” These data are expressed as mean ± SEM (*P* < 0.05).

We next examined the impact of IL-27p28 in human clinical forms of Chagas disease. To perform this, we stratified our Chagas disease sample population into controls and patients with mild, moderate, or severe heart dysfunction caused by *T. cruzi* infection. As suggested by the results in the experimental model of *T. cruzi* infection, serum levels of IL-27p28 were higher in patients with indeterminate form or mild heart dysfunction compared to chronic patients with moderate or severe heart dysfunction (Figure 9). These data indicate that IL-27p28 can also be used as a biomarker associated with different clinical forms of CCC.

### Polymorphic Sites at the *Ebi3* Gene Are Associated with Severe Cardiopathy in Chagas Disease

To confirm the importance of IL-27 in Chagas disease and understand whether polymorphic sites at *Ebi3* gene might contribute to the severity of the disease, we assessed whether the two most prevalent polymorphic regions at the *Ebi3* gene were related to the clinical manifestations of Chagas disease. Our cohort consisted of 395 individuals divided into two different groups: 93 indeterminate patients and 302 CCC patients. The indeterminate group included 31 (33.3%) males and 62 (66.7%) females, whereas the CCC group included 150 (49.7%) males and 152 (50.3%) females. Sex ratio was significantly different between the two groups (*p* = 0.006). The CCC patients were further divided into two subgroups: 174 patients with moderate cardiopathy (EF > 0.4) and 128 with severe cardiopathy (EF ≤ 0.4). The moderate cardiomyopathy subgroup included 67 (38.3%) males and 108 (61.7%) females, whereas the severe cardiomyopathy subgroup included 83 (65.4%) males and 44 (34.6%) females. Sex ratio was also significantly different between the two subgroups (*p* = 5E−6). Thus, we included sex in the analysis as a covariate.

Regarding the polymorphism at the position +609 of Ebi3 gene (SNP rs4740), we noticed that the genotype AA is less frequent in patients with severe cardiopathy (6.6%) compared to patients with the indeterminate form (19.8%) or those with moderate cardiopathy (20.2%) (Table 1). The following comparisons were done for SNP rs4740: CCC vs indeterminate, severe CCC vs indeterminate, and moderate CCC vs severe CCC. We compared patients carrying AA genotype to those carrying either GG or GA genotypes. By comparing CCC vs indeterminate patients, none of the covariates (sex and polymorphism) remained significantly associated with disease [sex: *p* = 0.836; OR (95% CI) = 0.933 (0.49–1.80); rs4740: *p* = 0.501; OR (95% CI) = 0.785 (0.39–1.59)]. By comparing severe CCC vs indeterminate patients, only the rs4740 polymorphism remained significantly associated [sex: *p* = 0.083; OR (95% CI) = 2.043 (0.91–4.58); rs4740: *p* = 0.025; OR (95% CI) = 3.157 (1.15–8.64)] after a multivariate binary regression analysis. By comparing moderate CCC vs severe CCC, the two covariates (sex and polymorphism) remained significantly associated with disease [sex: *p* = 0.004; OR (95% CI) = 2.833 (1.39–5.79); rs4740: *p* = 0.006; OR (95% CI) = 3.645 (1.45–9.16)]. Thus, this polymorphism distinguishes moderate CCC from severe CCC and severe CCC from asymptomatic controls.

**Table 1:** The inheritance of polymorphisms rs4740 and rs4905 at Ebi3 gene is associated with more severe cardiopathy in Chagas patients.

	Indeterminate group		CCC group		Moderate cardiomyopathy EF ≤ 0.4		Severe cardiomyopathy EF > 0.4	
Polymorphism	*n*	Frequency (%)	*n*	Frequency (%)	*n*	Frequency (%)	*n*	Frequency (%)
**Ebi3 + 609G > A (rs4740)**								
Alleles								
G	98	60.5	335	62.3	187	57.4	148	69.8
A	64	39.5	223	37.7	139	42.6	64	30.2
Total	162	100	538	100	326	100	212	100
Genotypes								
GG	33	40.7	106	39.4	57	35.0	49	46.2
GA	32	39.5	123	45.7	73	44.8	50	47.2
AA	16	19.8^a^	40	14.9	33	20.2^a^	7	6.6^a^
Total	81	100	269	100	163	100	106	100
**Ebi3 + 680 A > G (rs4905)**								
Alleles								
A	98	58.3	340	60.4	186	56.0	154	67.0
G	70	41.7	222	39.6	146	44.0	76	33.0
Total	168	100	562	100	332	100	230	100
Genotypes								
AA	34	39.3	104	37.0	55	33.1	49	42.6
AG	32	38.1	132	47.0	76	45.8	56	48.7
GG	19	22.6^a^	45	16.0	35	21.1^a^	8	8.7^a^
Total	84	100	281	100	166	100	115	100

*CCC, chronic Chagas cardiomyopathy; EF, ejection fraction*.

*^a^Statistically significant*.

Regarding the polymorphism at the position +680 of Ebi3 gene (SNP rs4905), we found that the genotype GG is also less frequent in patients with severe cardiopathy (8.7%) compared to the other groups (22.6% for indeterminate and 21.1% for moderate cardiopathy) (Table 1). The following comparisons were also done for SNP rs4905: CCC vs indeterminate, severe CCC vs indeterminate, and moderate CCC vs severe CCC. We compared subjects carrying GG genotype to those carrying either AA or AG genotypes. By comparing CCC vs indeterminate patients, only the sex remained significantly associated with the disease [sex: *p* = 0.008; OR (95% CI) = 0.499 (0.30–0.83)]. By comparing severe CCC vs indeterminate patients, both sex and polymorphism were associated with the disease [sex: *p* = 1.4E−5; OR (95% CI) = 3.800 (2.08–6.94); rs4905: *p* = 0.025; OR (95% CI) = 2.699 (1.13–6.43)] after a multivariate binary regression analysis. By comparing moderate CCC vs severe CCC, the two covariates remained significantly associated with disease [sex: *p* = 3.200E−5; OR (95% CI) = 2.899 (1.76–4.79); rs4905: *p* = 0.018; OR (95% CI) = 2.536 (1.18–5.47)]. This polymorphism also distinguishes moderate CCC from severe CCC and severe CCC from asymptomatic controls. Our data show that individuals who inherit the genotype AA for the rs4740 polymorphism or the GG for the rs4905 polymorphism have a lower risk to develop severe CCC. In our whole cohort, among the 135 individuals carrying the rs4740GG genotype, 131 of them also carry the rs4905AA genotype, suggesting that these regions are in linkage disequilibrium.

## Discussion

The plasticity of CD4 T cells contributes to the generation of a broad spectrum of inflammation. Th1 and Th17 cells seem to deviate this spectrum toward different directions. It was previously shown that CD4 T cells of IL-17A- and IL-23p19-deficient mice are shifted toward a Th1 profile after *T. cruzi* infection (28). We took advantage of this concept to overactivate Th1 cells by using genetically deficient mice for Th17-related molecules. These animals rapidly succumbed to the infection, most likely due to an inflammatory environment promoted by the hyperproduction of IFN-γ in their heart tissues, leading to tissue damage. IL-27p28 emerged as an important molecule in this system, which encouraged us to evaluate its suppressive functions during the *T. cruzi*-induced myocarditis.

IL-27 is thought to be a key regulator of the liver inflammation by suppressing IFN-γ-producing CD4 T cells caused by different species of *Trypanosoma* (23–25). Here, we showed that Ebi3, a subunit of IL-27, is not only important to ameliorate liver immunopathology, but more broadly it is also essential to suppress myocarditis caused by *T. cruzi*. In our model, it was evident that Ebi3 was important not only to suppress the immune response locally or systemically but it was also required to prevent tissue damage. It not only corroborates the importance of Ebi3 as a regulator of liver immunopathology but it also points out the importance of Ebi3 to modulate myocarditis upon *T. cruzi* infection. Although Ebi3 has a protective effect upon *T. cruzi* infection, the low levels of IL-12p35 cannot rule out a potential implication of IL-35 in this context, since IL-12, a very important cytokine associated with cardiac inflammation, would also be ruled out. In summary, Ebi3 has a broad spectrum of suppressive functions that help to maintain tissue integrity, even during the chronic stage of Chagas disease.

Our study also reveals that Ebi3 is a central modulator of inflammatory CD4 T cell subpopulations (16, 35, 36), especially by suppressing IFN-γ-producing Th1 cells. Even though IFN-γ restricts the parasite growth, its abundant production may lead to tissue damage and heart dysfunction (37) when the disease reaches its chronicity. Although Th1 lymphocytes are necessary to control the parasite growth *via* NO production (4, 5), an uncontrolled, exacerbated Th1 cell inflammation provokes severe *T. cruzi*-induced myocarditis (37). Our findings suggest that myocardial damage was mediated by IFN-γ-producing CD4^+^ T cells and, to a certain extent, by CD8 T cells. Th1 and CD8 T cells were suppressed by Ebi3 in the heart tissue. Moreover, inhibition of inflammatory mediators, such as IFN-γ or NO, in Ebi3-deficient mice was crucial to prevent host death after *T. cruzi* infection, supporting the hypothesis that Ebi3 has a suppressive impact on the inflammatory effects of IFN-γ and, consequently, has a fundamental role for host survival. These data are in accordance with others showing that NO down-modulates Th1 accumulation (38). Therefore, Ebi3 emerges as a novel regulatory candidate that negatively modulates the effects of IFN-γ in the cardiac tissue during experimental *T. cruzi* infection.

Suppressive effects of Ebi3 on other CD4 T cell subpopulations, such as Th17 and Th2 cells, have also been documented, albeit in the spleen of infected mice (25). Th1, Th2, and Th17 cytokines were augmented in the spleen of Ebi3-deficient mice during the acute phase of the infection with *T. cruzi* (7 d.p.i.), whereas the production of these inflammatory mediators in the spleen was comparable during the chronic phase of the infection (14 d.p.i.) (25). In our model, we found a pronounced production of inflammatory mediators in the heart of infected animals during the chronic phase of the disease (15 d.p.i.). We believe that the continuous presence of the parasite in the bloodstream during the acute phase promotes the production of inflammatory cytokines in the spleen, which corroborates the increased levels of Th1, Th2, and Th17 cytokines found in the spleen at this stage (25). Once the parasites leave the bloodstream and mainly accumulate in the heart and liver, effector immune cells migrate to these target organs and locally produce inflammatory mediators during the chronic phase of the disease, which supports the higher levels of inflammatory mediators found in the heart and liver 14–15 d.p.i., whereas comparable levels of cytokines are observed in the spleen at this time point. The absence of Ebi3 or IL-27Rα was also associated with a higher parasitemia after the experimental infection with 500 forms of the Tulahuen strain of *T. cruzi* (25). However, our study found a lower parasitemia in Ebi3-deficient mice after infection with 1,000 forms of the Y strain of *T. cruzi*. The differences in the parasitemia are most likely due to the differences in the strains and number of forms used to infect the animals in these studies.

Ebi3 was previously associated with inhibition of alternative macrophages in the spleen upon *T. cruzi* infection (25). The production of IL-4 and IL-13 was higher in spleen cells of infected Ebi3-deficient mice on day 7 post-infection, which probably generated an ideal microenvironment required to the differentiation of alternatively activated macrophages. Indeed, expression of *Arg-1* and *Chi3l3* encoding Ym1, markers of alternatively activated macrophages, was elevated in the spleen of Ebi3-deficient mice on day 14 after *T. cruzi* infection. In addition, neutralization of IL-4 in IL-27Rα-deficient mice (24) or inhibition of Arginase-1 in Ebi3-deficient mice (25) was essential to control the parasitemia, but did not alter the host survival. In our model, Ebi3 could not modulate the expression of IL-4 and IL-13 (key cytokines associated with alternative macrophages) and *Stat6*, a transcription factor that induces Ym1 expression on macrophages (39), or genes encoding *Arginase I, Ym1*, and *Fizz1* in the heart of infected mice. These findings suggest that Ebi3 can regulate alternatively activated macrophages in the spleen, but not in the heart of *T. cruzi*-infected animals. A better characterization of alternative macrophages and their functionality upon *T. cruzi* infection is indispensable. Overall, the absence of Ebi3 during the infection with *T. cruzi* led to the production of Th2 cytokines, which promoted parasite growth, and the induction of Th1 and Th17 immune responses, which in turn aggravated the inflammation in the liver and heart of infected animals.

Understanding the involvement of anti-inflammatory molecules upon Chagas disease is critical to design new therapeutic approaches. Despite anti-inflammatory mediators restrict liver and heart inflammation, the fact that they suppress important cell populations required to eliminate the parasite remains as a barrier to overcome. IL-27 was first described as a key mediator of Th1 lymphocytes due to its capacity to activate the JAK–STAT signaling pathway, which leads to STAT1 phosphorylation and ultimately T-bet activation and expression. This cascade of events promotes the production of IFN-γ (17). Recently, however, after exploring exhaustively its function, IL-27 has emerged as a potent anti-inflammatory mediator able to induce Tr1 cells, a non-classical regulatory CD4 T cell subpopulation that produces simultaneously IFN-γ and IL-10 (16, 18, 40). Since a fine tune balance between parasite control and immune response is a major key factor to prevent *T. cruzi*-associated pathogenesis, IL-27 could be a strategic molecule to control both the immune response and the parasite growth due to its dual role either acting as a suppressor molecule or an inductor of IFN-γ.

Although several efforts are still needed to understand the pathways involved in the ability of *T. cruzi* to induce IL-27 production, our findings suggest that a myeloid cell population is the main source of IL-27p28 production. Phenotypically, the IL-27p28-producing myeloid cells expressed CD11b and MHCII. A better characterization of this population in the heart is difficult, since this tissue is very compact and the isolation of immune cell populations requires a harsh treatment with proteolytic enzymes that kill the majority of the cells. Macrophages and dendritic cells are the main sources of IL-27 production during infectious and autoimmune diseases (14, 15). Dendritic cells are thought to be the main source of IL-27 in non-infectious conditions, such as autoimmune diseases and cancer. The IL-27 may autocrinely act on DCs to promote the expression of CD39 on their surface. CD39-expressing DCs converted extracellular ATP to ADP and AMP molecules, which were critical to down-regulate NLRP3 inflammasome and protect the host from experimental autoimmune encephalomyelitis (EAE) (41). Also, IL-27-releasing DCs induced a proper microenvironment for cancer growth, since IL-27 promoted CCL22 production and consequently mediated the recruitment of Tregs to the tumor site (42). In our system, myeloid cells were able to respond *in vitro* to the challenge with *T. cruzi* and produce IL-27p28. Myeloid cells were described as primary cellular sources of IL-27p28 in the heart tissue during the experimental *T. cruzi* infection, although further characterization is needed.

Recent data have increasingly shown the signaling pathways for IL-27 production and its gene interrelation, indicating its role as pro- and anti-inflammatory cytokine (16–18). The production of p28 subunit is dependent on the TLR4-associated MyD88-mediated pathway, besides the transcription factors NFκB, c-Rel, AP-1, and c-Fos (35). TLR4 can induce the expression of p28 through activation of the TIR domain-containing adaptor inducing IFN-β (TRIF) and IFN regulatory factor 3 (IRF3) pathways (35). TLR2-, TLR4-, and TLR9-associated MyD88 are required for the induction of Ebi3 expression through binding of NF-kB subunits (p50/p65) and PU.1 to the Ebi3 promoter. IFN-γ-induced IRF-8 expression can upregulate p28 gene transcription in synergy with IRF-1 (35). In the context of infection with *T. cruzi*, it was not clear what sensing pathways were strictly involved in the IL-27 production. It is known that several innate immune sensors on the macrophage membrane recognize *T. cruzi* molecules or danger signals released by the *T. cruzi*–host interaction (30–32). TLR-2 (30), 4 (31), and 9 (32) are well-characterized receptors that sense *T. cruzi* to induce effector or regulatory mediators. Besides, triggering TLR4 with LPS induced the production of IL-27 by monocytes and macrophages (43). Based on their fundamental role in *T. cruzi* recognition, we showed that TLR2 and TLR4 sensors play a pivotal role for the optimal production of IL-27p28 without interfere in the macrophage parasitism. Our data show that it is necessary co-trigger TLR2 and TLR4 to induce IL-27p28, although the absence of TLR4 alone abrogates the *T. cruzi*-induced IL-27p28 production in 87.8%. Both receptors had an additive effect on IL-27p28 production during *T. cruzi* recognition.

The proposed paradigm for the broad spectrum of CCC is determined by an imbalance of the cytokine production (44). It is postulated that an anti-inflammatory cytokine profile, mainly induced by IL-10 signaling, does not compromise the cardiac tissue of patients and leads to the indeterminate form of the disease, while the cardiac form is induced and maintained by an inflammatory cytokine profile, where IFN-γ and TNF-α play a pivotal role (44). Indeed, a polymorphic site at *Il10* gene was associated with lower levels of IL-10 and development of CCC (45). In addition, T cells from indeterminate patients have higher expression of the inhibitory receptor CTLA-4 than patients with severe cardiac forms of the disease (46). Besides, monocytes from indeterminate patients produced high levels of the anti-inflammatory cytokine IL-10 and higher IL-10/TNF-α ratio, whereas those from CCC patients displayed high levels of the pro-inflammatory cytokine TNF-α (46, 47). These pieces of evidence reveal a predominance of an anti-inflammatory environment required to mitigate a deleterious immune response in indeterminate forms, while an inflammatory environment, mediated by IFN-γ and TNF-α, is predominant in the cardiac forms of Chagas disease and leads to tissue damage and dysfunction (44).

Understanding the mechanism by which IL-27 exerts its suppressive effects on Th1 cells is extremely important to devise strategies capable of controlling *T. cruzi* infection. IL-27 suppresses Th1 immune response by reducing IL-2 production (22). This has a direct effect on Th1 cells, which are polarized to classical IL-10-producing CD4^+^Foxp3^+^ T cells or non-classical CD4^+^Foxp3^−^ Tr1 cells (40). In a study of human visceral leishmaniasis, pro-inflammatory cytokines acting on splenic macrophages had the potential to upregulate IL-27, which in turn induced IL-21 to expand IL-10^+^CD4^+^CD25^−^Foxp3^−^ T cells as a mechanism of positive feedback control (48). During *T. cruzi* infection, IL-10 is crucial to modulate the pathogenic immune response and prevent its consequences associated with tissue damage. CD4 T cells are the main producers of IL-10, whose actions counterbalance pathogenic cytokines, such as IL-12, TNF-α, and IFN-γ (49, 50). In our system, Ebi3 could not induce classical IL-10-producing Foxp3^+^ Tregs (51), but instead it triggered the induction of IL-10-producing Foxp3^−^ Tr1 cells in the cardiac tissue of infected mice. Böhme et al. found a subtle reduction in the frequency of Foxp3^+^CD25^+^CD4^+^ cells in the spleen of Ebi3-deficient mice at day 7 post-infection, which was no longer significant at day 14 post-infection. Also, no differences in the absolute number of Tregs were detected in the spleen of WT and Ebi3-deficient mice across the time points. In addition, the functionality of these cells, either by their capacity to produce anti-inflammatory mediators or their ability to suppress inflammatory cells, was not analyzed (25). Besides, Ebi3 was essential to regulate the production of IFN-γ by CD4 T cells, as demonstrated here and elsewhere using another species of *Trypanosoma* (23), and to a lesser extent by CD8 T cells. Indeed, the inhibition of IFN-γ at later time points ameliorated the survival of infected Ebi3-deficient mice. Our group showed that the regimens of anti-IFN-γ-treatment only have an impact on the parasite growth when administrated in the beginning of the infection. The treatment at later time points failed to control the parasitemia (52). Collectively, these pieces of evidence reinforce that Tr1 cells, locally induced by Ebi3, protected the host from the inflammation caused by IFN-γ, thus highlighting a novel mechanism employed by Ebi3 to dampen *T. cruzi*-induced myocarditis *via* accumulation of Tr1 cells in the heart and suppression of IFN-γ.

Members of the IL-12 family have opposite effects on the immunity, depending on how they are formed as heterodimers, since they share common subunits. IL-12, IL-23, and IL-39 promote inflammatory immune responses through different mechanisms, whereas IL-27, IL-30, and IL-35 induce suppressive responses. IL-27, our main focus, shares the Ebi3 subunit with IL-35 and IL-39, whereas the IL-27p28 subunit may act as IL-30. Despite entirely focusing our analysis on Ebi3 and IL-27p28, we cannot rule out the importance of the other anti-inflammatory members of IL-12 family, such as IL-30, IL-35 and IL-39, upon *T. cruzi* infection. Even though the gene expression of the p35 subunit was very low in the heart tissue at day 9 post-infection, which was not analyzed in the late time-points, where inflammation reaches its peak. We also do not discard the possibility of IL-27p28 to solely act as a suppressive molecule during *T. cruzi* infection. Yet, the participation of the recently discovered IL-39, composed of Ebi3 and IL-23p19, two subunits that play a role during *T. cruzi* infection (28), cannot be ruled out based on our findings. Further characterization is needed to determine whether IL-30, IL-35, and IL-39 can also mitigate the myocarditis induced by *T. cruzi*.

Patients with severe CCC commonly develop arrhythmias, heart failure, thromboembolism, and sudden death (1). Novel molecular biomarkers that are correlated with the severity of CCC would be most welcome to improve treatment and prognosis of the disease. Some pieces of evidence found a correlation between IL-17 (29), IL-10 (53), and IFN-γ production (54) and the severity of the clinical forms of Chagas disease. In addition, recent studies have correlated IL-27 levels with the morbidity of multiple sclerosis (55), arthritis, and experimental models of disease mediated by Th17 cells (56). Here, we showed that IL-27 seems to have a protective role in Chagas disease. The presence of IL-27p28 in the serum of the patients was indicative of better CCC prognosis. Indeed, polymorphic sites at Ebi3 gene in Chagas disease patients were associated with more severe forms of CCC, suggesting that IL-27 may have a protective effect on the pathogenesis of the human disease. Although higher levels of IL-27 are associated with higher cardiac inflammation in the absence of Th17-related molecules, we believe that the impairment in the Th17 response naturally polarizes CD4 T cells toward a pathogenic IFN-γ-producing T cell population when primed by *T. cruzi*. In the absence of Th17-related molecules, this population accumulates and causes damages in the heart tissue in the early stage of the disease. We believe that suppressive mediators, such as IL-27, are secreted in the later stage of the disease as an attempt of counterbalancing the pathogenic IFN-γ-producing CD4 T cells rather than being a causative factor directly associated with the severity of myocarditis. On the contrary, in humans, we believe that high levels of IL-27 could control the cardiac inflammation most likely driven by a cardiac accumulation of IFN-γ, as CD4 T cells were not forcedly polarized toward a hyperactive Th1 profile in this context. Therefore, the severity of the myocarditis is due to an accumulation of pathogenic IFN-γ-producing CD4 T cells rather than the higher production of IL-27.

Collectively, our data support Ebi3 as a novel regulatory immune-modulator that prevents cardiomyopathy, thus opening a reasonable perspective for the therapeutic manipulation of Ebi3 that could be useful for patients with Chagas disease.

## Ethics Statement

This study was carried out in accordance with the recommendations of the Institutional Ethics Committee Hospital das clinicas de Ribeirão Preto—USP, São Paulo, Protocol number 2285/2007; Brazil and the Institutional Ethics Committee–CAPpesq–HC-FMUSP—Protocol Number 0265/10, Brazil with written informed consent from all subjects. All subjects gave written informed consent in accordance with the Declaration of Helsinki. The protocol was approved by the Institutional Ethics Committee Hospital das clinicas de Ribeirão Preto—USP, São Paulo. This study was carried out in accordance with the recommendations of the Institutional Animal Care and Use Committee (IACUC)/Ethics Committee for Animal Care and Research (CETEA-FMRP/USP) under protocol 192/2011. The protocol was approved by the National Committee for Control of Animal Research (CONCEA).

## Author Contributions

Conceived and designed the experiments: TM and JS. Performed the experiments: TM, GO, MS, BD, GS, DF, and RC. Analyzed the data: TM, GO, and MS. Writing—original draft: TM. Writing—review and editing: TM, JS, JN, EN, GO, MS, and CC. Statistics: TM, GO, CC, and EN. Patient’s recruitment, collection of human samples or DNA extraction: AF, MB, BI, and AP.

## Conflict of Interest Statement

The authors declare that the research was conducted in the absence of any commercial or financial relationships that could be construed as a potential conflict of interest.
